# The Role of Contrast-Enhanced Ultrasound in the Differential Diagnosis of Malignant and Benign Subpleural Lung Lesions

**DOI:** 10.3390/jcm13082302

**Published:** 2024-04-16

**Authors:** Andrea Boccatonda, Maria Teresa Guagnano, Damiano D’Ardes, Francesco Cipollone, Luigi Vetrugno, Cosima Schiavone, Fabio Piscaglia, Carla Serra

**Affiliations:** 1 Internal Medicine, Bentivoglio Hospital, AUSL Bologna, 40010 Bentivoglio, Italy; 2 Department of Medical and Surgical Sciences, University of Bologna, 40138 Bologna, Italy; fabio.piscaglia@unibo.it; 3 Department of Medicine and Aging Science, Institute of “Clinica Medica”, “G. d’Annunzio” University of Chieti, 66100 Chieti, Italy; guagnano@unich.it (M.T.G.); francesco.cipollone@unich.it (F.C.); 4 Department of Anesthesiology, Critical Care Medicine and Emergency, SS. Annunziata Hospital Chieti, 66100 Chieti, Italy; luigi.vetrugno@unich.it; 5 Internistic Ultrasound Unit, SS Annunziata Hospital, “G. d’Annunzio” University, 66100 Chieti, Italy; cosima.schiavone@gmail.com; 6 Division of Internal Medicine, Hepatobiliary and Immunoallergic Diseases, IRCCS Azienda Ospedaliero—Universitaria di Bologna, 40138 Bologna, Italy; 7 Interventional, Diagnostic and Therapeutic Ultrasound Unit, IRCCS Azienda Ospedaliero—Universitaria di Bologna, 40138 Bologna, Italy; carla.serra@aosp.bo.it

**Keywords:** lung, CEUS, consolidation, ultrasound, malignant lesion

## Abstract

**Background:** The application of transthoracic contrast-enhanced ultrasound (CEUS) to the study of peripheral lung lesions is still a topic of debate. The main objective of this review was to evaluate the diagnostic accuracy of CEUS in the diagnosis of malignant subpleural pulmonary consolidations and, therefore, differentiate them from benign ones. **Methods:** Papers published before December 2023 were detected through a search of PubMed, Cochrane library, and Embase. The pooled specificity and sensitivity, summary receiver operating characteristic (SROC) curve and diagnostic odds ratio (DOR) were used. **Results:** CEUS is characterized by a pooled sensitivity of 0.95 (95% CI: 0.93–0.97) and a pooled specificity of 0.93 (95% CI: 0.90–0.95) in differentiating benign and malignant subpleural lung diseases; the AUC of SROC was 0.97. Homogeneous CE was characterized by a pooled sensitivity of 0.43 (95% CI: 0.40–0.45) and the pooled specificity of 0.49 (95% CI: 0.46–0.52). Non-homogeneous CE displayed a pooled sensitivity of 0.57 (95% CI: 0.55–0.60) and a pooled specificity of 0.51 (95% CI: 0.48–0.54). The lack of CE displayed a pooled sensitivity of 0.01 (95% CI: 0.00–0.06) and a pooled specificity of 0.76 (95% CI: 0.64–0.85). Marked CE displayed a pooled sensitivity of 0.41 (95% CI: 0.37–0.44) and a pooled specificity of 0.54 (95% CI: 0.50–0.58). Non-marked CE displayed a pooled sensitivity of 0.59 (95% CI: 0.56–0.63) and a pooled specificity of 0.46 (95% CI: 0.42–0.50). The early AT displayed a pooled sensitivity of 0.04 (95% CI: 0.02–0.08) and a pooled specificity of 0.83 (95% CI: 0.77–0.87). The early wash out displayed a pooled sensitivity of 0.61 (95% CI: 0.48–0.72) and a pooled specificity of 0.98 (95% CI: 0.92–1.00). The delayed wash out displayed a pooled sensitivity of 0.15 (95% CI: 0.10–0.20) and a pooled specificity of 0.69 (95% CI: 0.62–0.75). **Conclusions:** CEUS is characterized by excellent diagnostic accuracy for the diagnosis of the malignancy of subpleural lung lesions. By separately analyzing the CEUS findings, the diagnostic accuracy values are considerably lower and not significant in some cases. The simultaneous evaluation of multiple CEUS features allows us to reach an excellent diagnostic accuracy. Non-homogeneous CE with early wash out are the most indicative features of malignancy of a lung lesion.

## 1. Introduction

The evaluation of pulmonary consolidations is one of the fields of application of lung ultrasound [1]. Based on the physics of ultrasound, transthoracic lung ultrasound can highlight only peripheral pulmonary consolidations affecting the pleura; indeed, the interposition of just a minimal layer of air (aerated lung) between the lesion and the pleura generates air artifacts, called A lines, which obscure the visualization of the lesion in depth [2]. Lung consolidations can be of various natures such as atelectasis, infectious pneumonia, contusions, and neoplastic diseases [3,4]. In general, it is relevant to first differentiate a malignant lesion from a benign one. Some works in the literature have attempted to find differential findings using B-mode such as margins, echostructure, and the presence of bronchograms, but none have shown acceptable diagnostic accuracy alone or in association [5,6].

Even the ultrasound color Doppler study did not prove to be completely reliable in differentiating between benign and malignant lesions [7,8]. In many fields of ultrasound study, such as focal liver diseases, the application of contrast-enhanced ultrasound (CEUS) has found wide application, leading to the codification of a specific semiotics or score capable of diagnosing and differentiating malignant vs. benign pathologies with excellent diagnostic accuracy [1,9,10]. In general, fast and intense enhancement in the arterial phase and complete wash out are the typical contrastographic characteristics of malignant lesions, mainly due to tumor neoangiogenesis [1,11]. The application of CEUS to the study of lung lesions is still a topic of debate, with little evidence [12,13]. The concern is due to the presence of a double vascularization at the pulmonary level, which makes it difficult to apply of the “classic” contrastographic study schemes used in other organs [14].

The main objective of this systematic review and meta-analysis is to evaluate the diagnostic accuracy of CEUS in the diagnosis of malignant subpleural pulmonary consolidations and, therefore, differentiate them from benign ones. Second, we aim to evaluate the diagnostic accuracy of different contrastographic characteristics in the diagnosis of malignant lesions such as the presence or lack of contrast enhancement (CE), homogeneous or non-homogeneous CE, marked or non-marked CE, early arrival time (AT), and early or delayed wash out.

## 2. Material and Methods

### 2.1. Literature Search Strategy

Papers published before December 2023 were detected through a comprehensive search of PubMed, Embase, and Cochrane library. The search terms were combinations of the relevant medical subject heading (MeSH) terms, key words, and word variants for “lung”, “neoplasm”, and “contrast-enhanced ultrasound”. Each study’s title and abstract were examined first and then the complete text was read to further filter the publications. Furthermore, a manual screening of each article’s references was conducted to find other potentially relevant studies.

The eligibility of these papers was determined based on the following criteria. Additionally, a third reviewer arbitrated disputes and disagreements. In the event that more details were required, we contacted the authors.

This study has an associated PROSPERO registration number 509477.

### 2.2. Inclusion and Exclusion Criteria

Prior to the literature search, inclusion and exclusion criteria were established. Studies were chosen based on whether or not they met the following requirements: clinical studies focused on the diagnostic value of CEUS for differentiating between benign and malignant subpleural lesions of the lung; histopathological findings served as the gold standard for diagnosis; there were enough data to create a 2 × 2 contingency table for true positives (TP), false positives (FP), true negatives (TN), and false negatives (FN); each patient provided informed consent, which was approved by the ethics committee; the articles were written in English. Research that fit one of the following criteria was excluded: editorial articles, case reports, reviews, or any study with insufficient data.

### 2.3. Data Extraction

Two researchers worked independently to extract the following data: the identity of the first author, the study’s year of publication, the nation, the average patient age, the total number of patients and lesions, and the gold reference standard. False positive (FP), false negative (TN), true positive (TP), and false negative (FN) data were either directly gathered or computed based on sensitivity, specificity, positive predictive value (PPV), and negative predictive value (NPV) in each of the chosen studies. A third reviewer evaluated the divergences.

### 2.4. Quality Assessment

Two researchers independently employed the Quality Assessment of Diagnostic Ac-curacy Studies (QUADAS) tool, which consisted of 19 questions, to evaluate the methodological quality of the included studies. The study was graded as “yes” (high quality) for each item if it was reported; “no” (poor quality) if it was not; and “unclear” if insufficient information was given. A third researcher also helped to settle disagreements. Table 1 displays comprehensive data on sample size, age, gender, and the reference standard in each particular study.

### 2.5. Statistical Analysis

RevMan 5.0 and Meta-Disc Version 1.4 (Unit of Clinical Biostatistics team of the Ramony Cajal Hospital, Madrid, Spain) were used for all statistical analyses. From the TP, FP, FN, and TN of each study, a summary of the sensitivity, specificity, positive likelihood ratios (PLR), negative likelihood ratios (NLR), and diagnostic odds ratios (DOR) was calculated. These values showed the accuracy of CEUS in differentiating between benign and malignant subpleural lung lesions. To summarize the TP and FP rates, meanwhile, the summary receiver operating characteristics (SROC) curve described by Moses et al. was created (15). To find the heterogeneity between several studies, the inconsistency index (I2) was utilized. When I2 > 50% revealed considerable heterogeneity (16), we would proceed with our analysis using a random effects model. Publication bias was analyzed by a contour enhanced funnel plot; when *p* > 0.05, we considered the study to have no relevant publication bias [25].

## 3. Results

### 3.1. Study Identification

**Figure 1:**
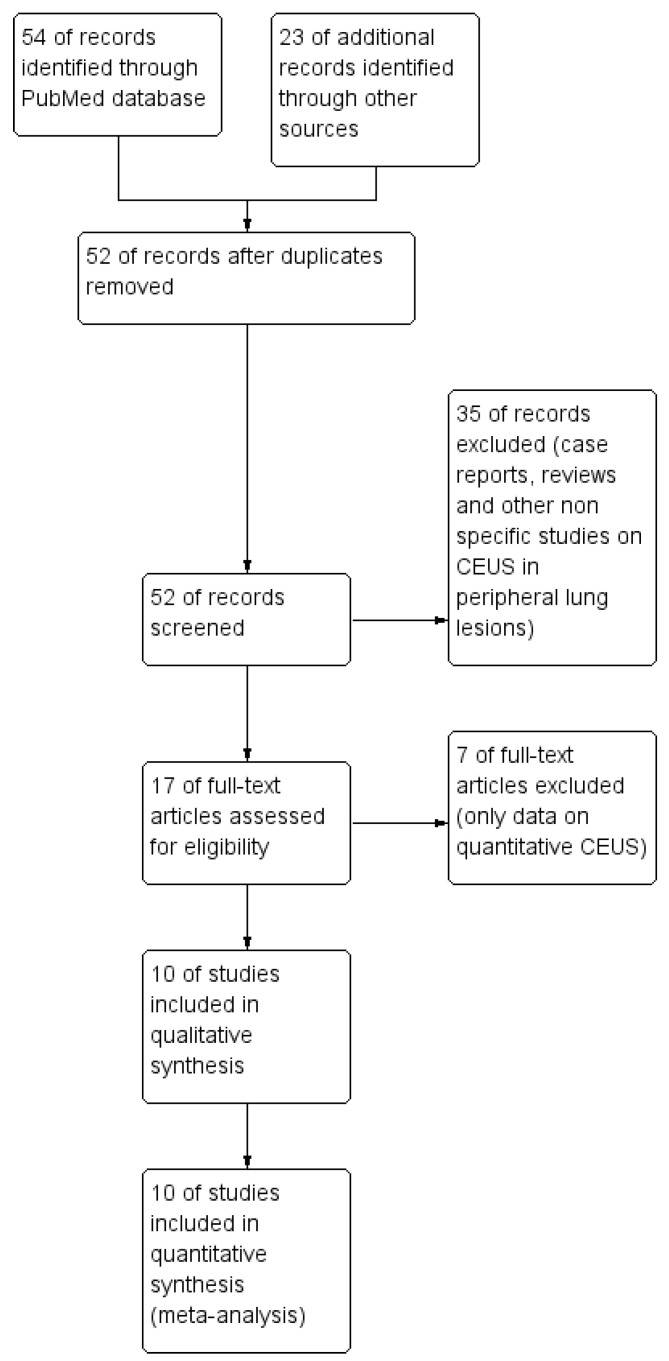
Study flow diagram.

A total of 52 relevant articles were detected in the initial search stage; many of these studies were excluded due to titles and abstracts. Only 17 studies were selected for full-text review. Further analysis excluded 7 studies lacking data on qualitative CEUS. Finally, 10 articles satisfying the inclusion criteria were included and analyzed [15,16,17,18,19,20,21,22,23,24]. The diagram of study search flow is reported in Figure 1.

### 3.2. Study Characteristics

Table 1 reports study characteristics concerning all included papers. Among these 10 selected studies, only 3 papers evaluated the overall diagnostic role of CEUS [16,17,22]. In total, 970 patients with 536 malignant lesions were analyzed. By dividing the CEUS study into the different phases and findings, homogeneous or non-homogeneous CE was evaluated in 8 studies for a total of 2623 patients and 1413 malignant lesions [15,16,17,18,19,20,21,23]; the lack of CE was evaluated in 2 studies on 158 patients with 92 malignant lesions [18,20]; marked or unmarked CE was evaluated in 4 studies on 1476 patients with 819 malignant lesions [16,18,20,21]; early AT was evaluated in 2 studies involving 412 patients with 200 malignant lesions [19,20]; early wash out was evaluated in 2 studies on 155 patients with 71 malignant lesions [19,20]; delayed wash out was evaluated in 3 studies on 472 patients with 218 lesions [17,19,20].

### 3.3. Quality Assessment

**Figure 2:**
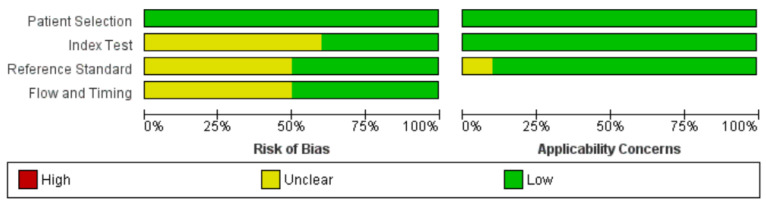
Risk of bias and applicability concerns graph: review authors’ judgements about each domain presented as percentages across included studies.

**Figure 3:**
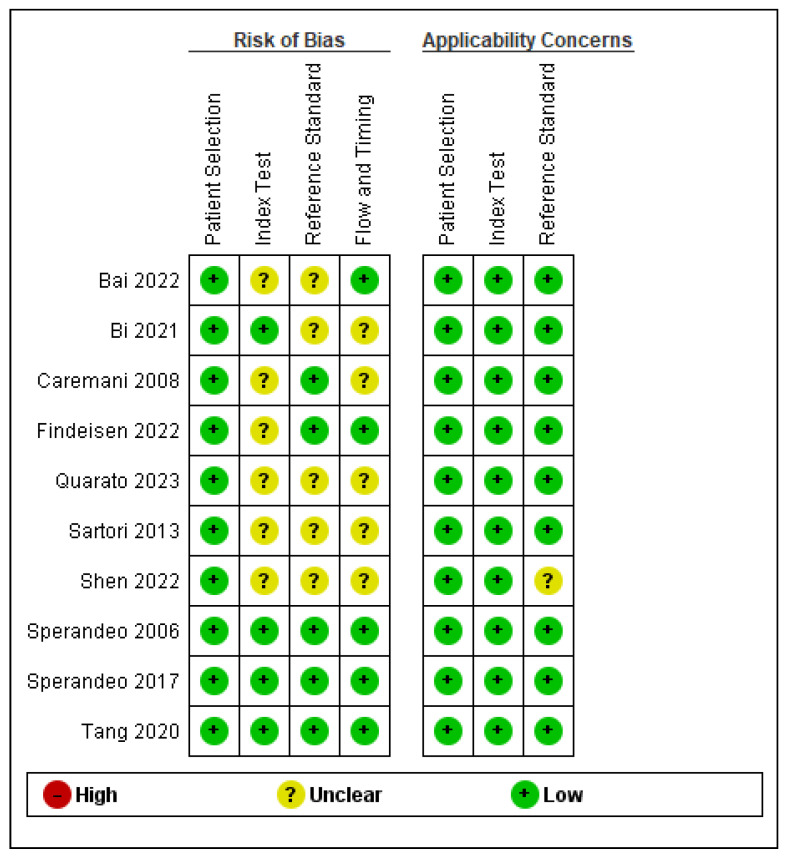
Risk of bias and applicability concerns summary: review authors’ judgements about each domain for each included study [15,16,17,18,19,20,21,22,23,24].

An unclear risk of bias in the item of “index test” has been noted because in 7 works, it is not clearly explained whether the methods were performed and interpreted in a blinded manner [14,15,16,17,19,20,21] (Figure 2 and Figure 3). Furthermore, an unclear risk of bias in the item of “reference standard” was observed because in 6 studies, not all patients underwent biopsy, and the diagnosis of malignancy or benignity was made on the basis of clinical follow-up and/or radiological evidence [16,17,19,20,21,23] (Figure 2 and Figure 3). Detailed information regarding sample size, age, gender, and reference standard in individual study are shown in Table 1.

### 3.4. Overall Diagnostic Accuracy of CEUS

**Figure 4:**
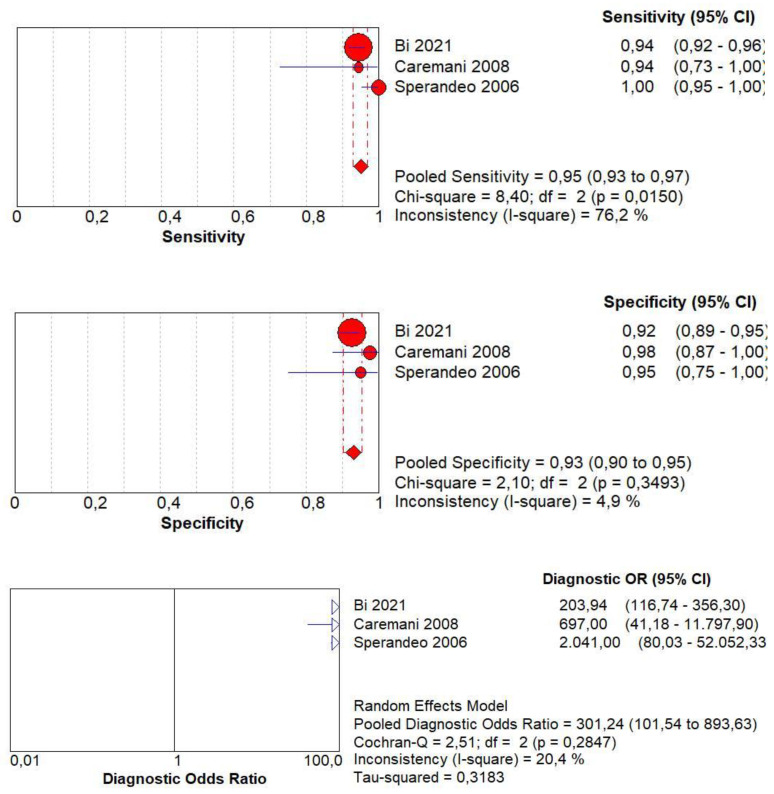

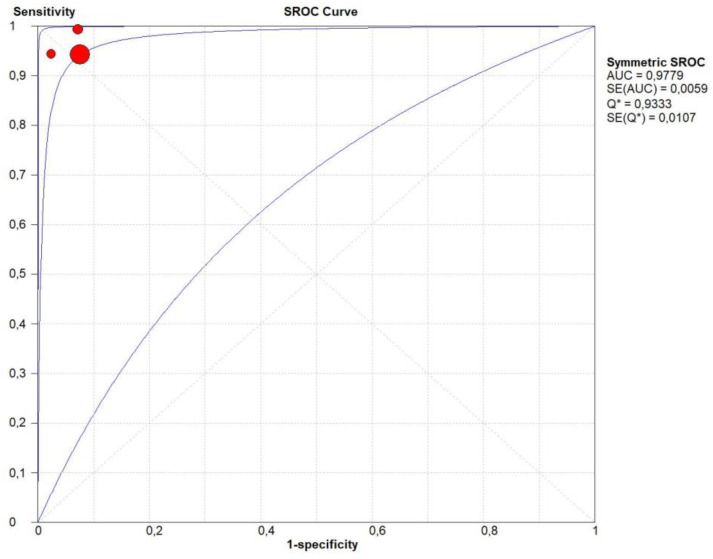
Sensitivity, specificity, diagnostic odds ratio, and SROC curve of CEUS in differentiating malignant vs. benign peripheral lung lesions. In particular, 970 patients with 536 malignant lesions were analyzed [15,17,22].

Concerning the diagnostic accuracy of CEUS in terms of differentiation between malignant and benign subpleural lung diseases, the pooled sensitivity was 0.95 (95% CI: 0.93–0.97) and the pooled specificity was 0.93 (95% CI: 0.90–0.95). The DOR was 301 (95% CI: 101–893). The AUC of SROC was 0.97. Significant heterogeneity was found in sensitivity (I^2^ = 76.2%), and no significant inconsistency was found in specificity (I^2^ = 4.9%) (Figure 4).

### 3.5. Diagnostic Accuracy of Homogeneous CE

**Figure 5:**
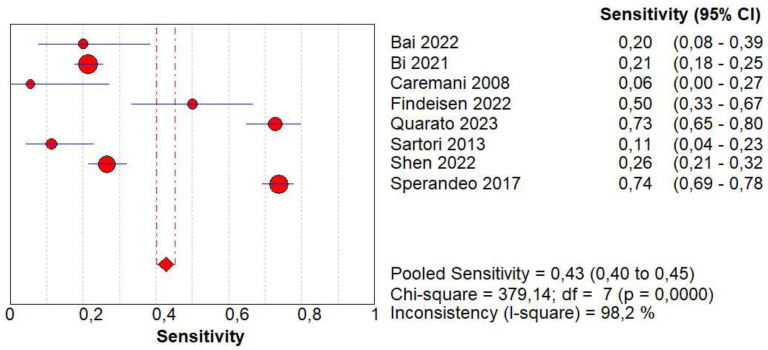

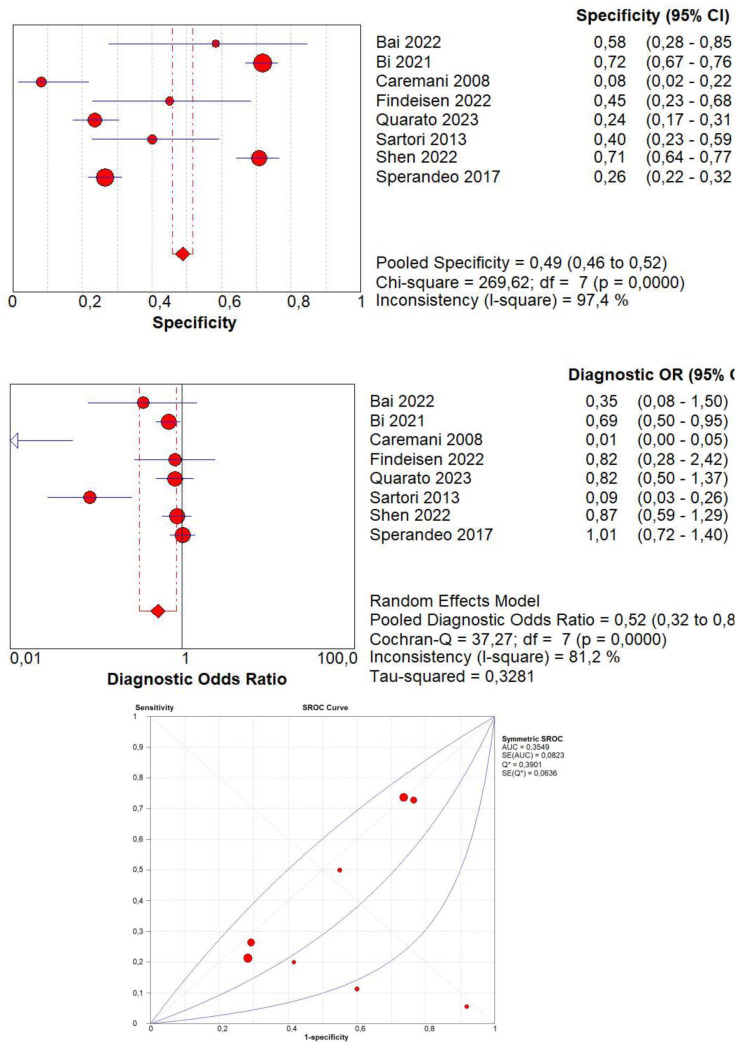
Sensitivity, specificity, diagnostic odds ratio, and SROC curve of homogeneous contrast enhancement in differentiating malignant vs. benign peripheral lung lesions. In particular, 8 studies for a total of 2623 patients and 1413 malignant lesions were examined [15,16,17,18,19,20,21,23].

Concerning the diagnostic quality of homogeneous CE in the differentiation between benign and malignant subpleural lung diseases, the pooled sensitivity was 0.43 (95% CI: 0.40–0.45), and the pooled specificity was 0.49 (95% CI: 0.46–0.52). The DOR was 0.52 (95% CI: 0.3–0.8). The AUC of SROC was 0.35. A significant heterogeneity was found in sensitivity (I^2^ = 98.2%), and a significant inconsistency was found in specificity (I^2^ = 97.4%) (Figure 5).

### 3.6. Diagnostic Accuracy of Non-Homogeneous CE

**Figure 6:**
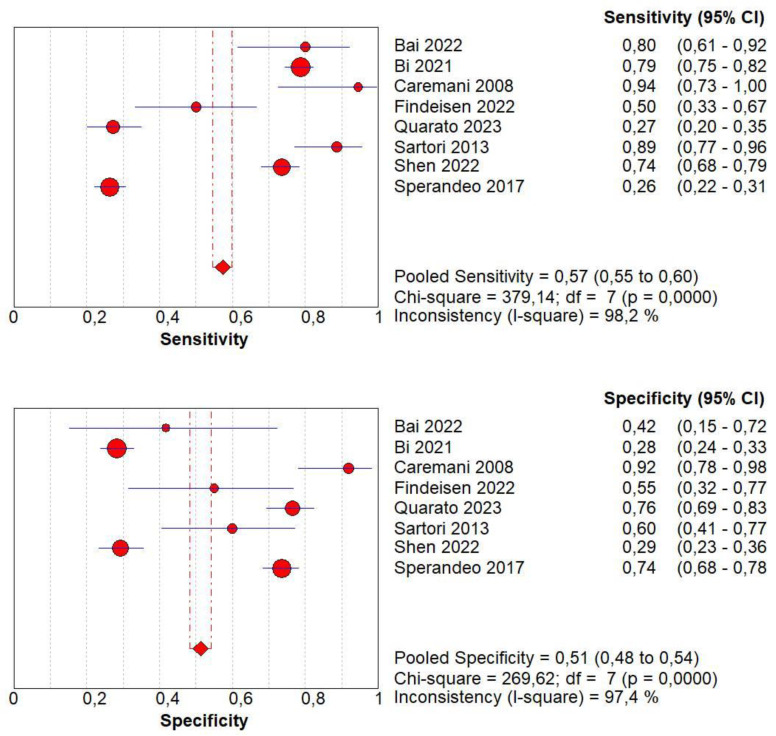

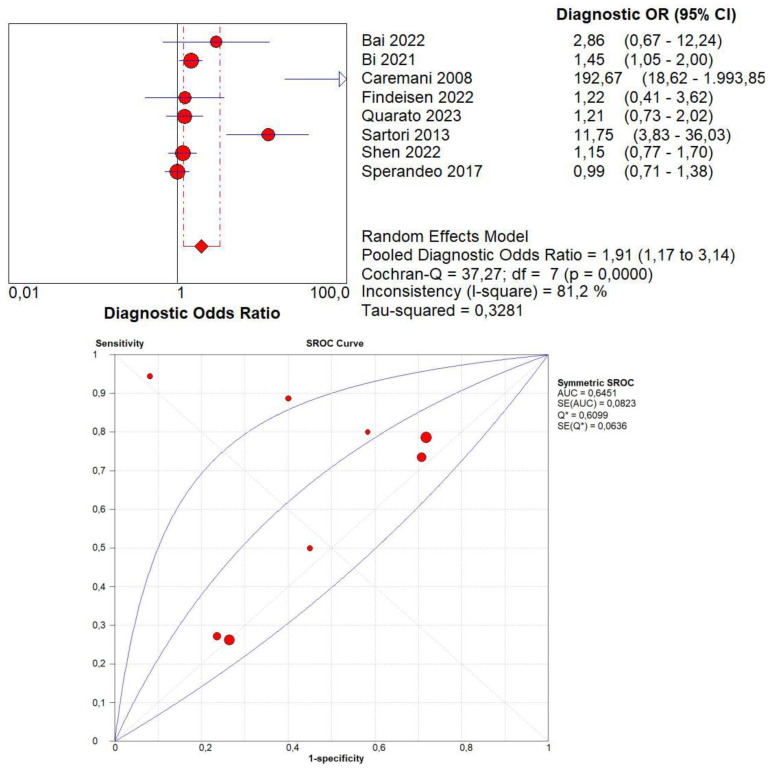
Sensitivity, specificity, diagnostic odds ratio, and SROC curve of non-homogeneous contrast enhancement in differentiating malignant vs. benign peripheral lung lesions. In particular, 8 studies for a total of 2623 patients and 1413 malignant lesions were examined [15,16,17,18,19,20,21,23].

Concerning the diagnostic quality of non-homogeneous CE in the differentiation between benign and malignant subpleural lung diseases, the pooled sensitivity was 0.57 (95% CI: 0.55–0.60), and the pooled specificity was 0.51 (95% CI: 0.48–0.54). The DOR was 1.91 (95% CI: 1.1–3.4). The AUC of SROC was 0.64. A significant heterogeneity was found in sensitivity (I^2^ = 98.2%), and a significant inconsistency was found in specificity (I^2^ = 97.4%) (Figure 6).

### 3.7. Diagnostic Accuracy of Lack of CE

**Figure 7:**
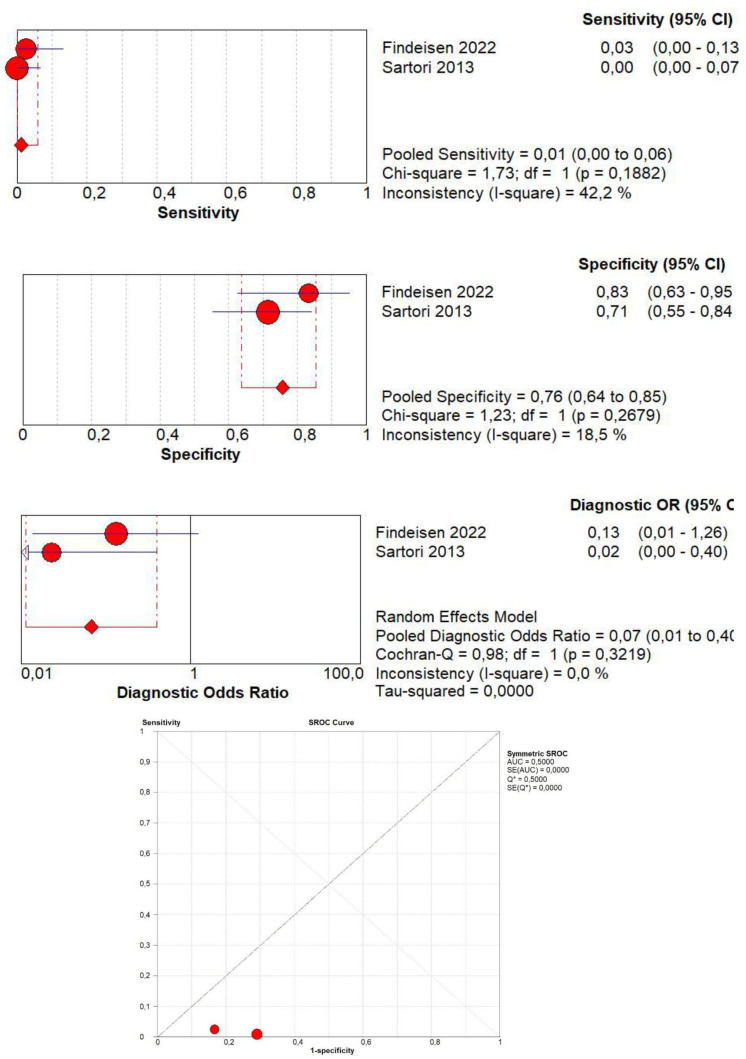
Sensitivity, specificity, diagnostic odds ratio, and SROC curve of a lack of contrast enhancement in differentiating malignant vs. benign peripheral lung lesions. In particular, 2 studies on 158 patients with 92 malignant lesions were included [18,20].

Concerning the diagnostic quality of a lack of CE in the differentiation between benign and malignant subpleural lung diseases, the pooled sensitivity was 0.01 (95% CI: 0.00–0.06), and the pooled specificity was 0.76 (95% CI: 0.64–0.85). The DOR was 0.07 (95% CI: 0.0–0.4). The AUC of SROC was 0.50. Concerning the lack of CE, a no significant heterogeneity was found in sensitivity (I^2^ = 42.2%), and a no significant inconsistency was found in specificity (I^2^ = 18.5%) (Figure 7).

### 3.8. Diagnostic Accuracy of Marked CE

**Figure 8:**
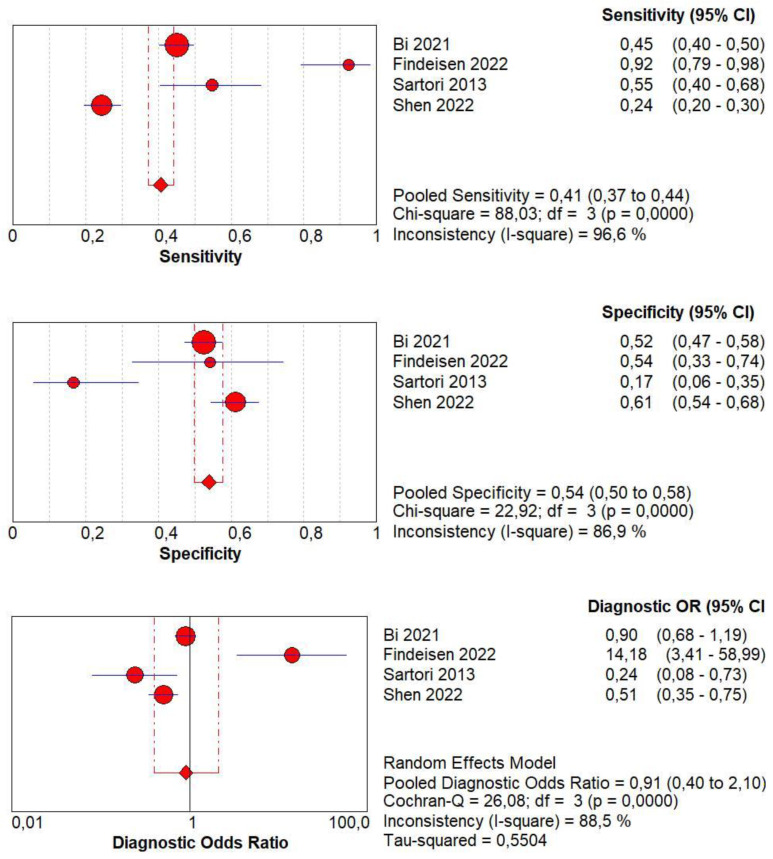

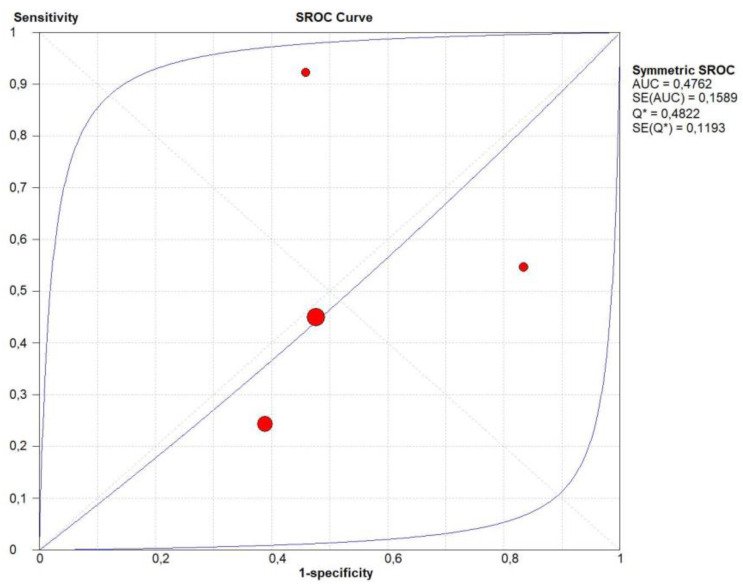
Sensitivity, specificity, diagnostic odds ratio, and SROC curve of marked contrast enhancement in differentiating malignant vs. benign peripheral lung lesions. In particular, 4 studies on 1476 patients with 819 malignant lesions were examined [16,18,20,21].

Concerning the diagnostic quality of marked CE in the differentiation between benign and malignant subpleural lung diseases, the pooled sensitivity was 0.41 (95% CI: 0.37–0.44), and the pooled specificity was 0.54 (95% CI: 0.50–0.58). The DOR was 0.91 (95% CI: 0.4–2.1). The AUC of SROC was 0.47. A significant heterogeneity was found in sensitivity (I^2^  = 96.6%), and a significant inconsistency was found in specificity (I^2^ = 86.9%) (Figure 8).

### 3.9. Diagnostic Accuracy of Non-Marked CE

**Figure 9:**
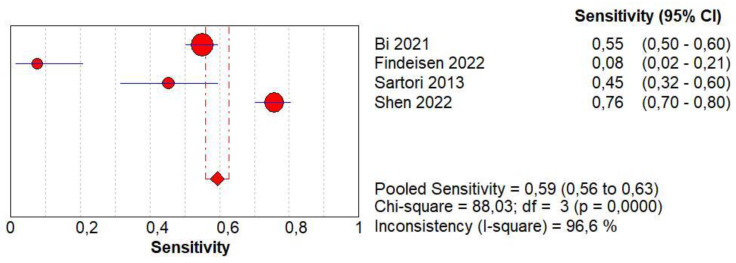

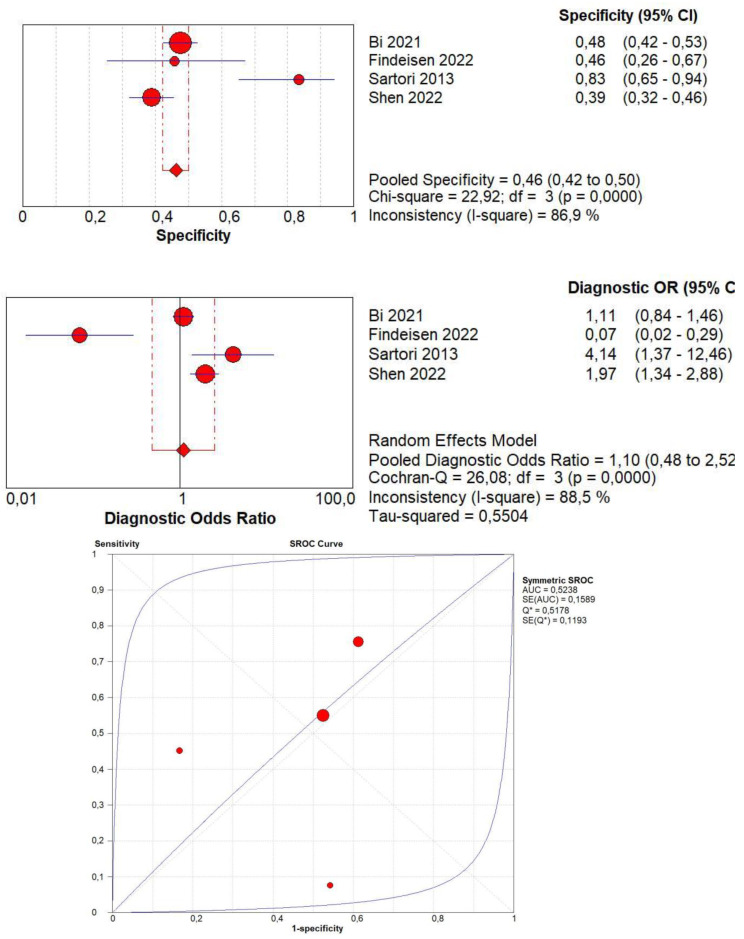
Sensitivity, specificity, diagnostic odds ratio, and SROC curve of non-marked contrast enhancement in differentiating malignant vs. benign peripheral lung lesions. In particular, 4 studies on 1476 patients with 819 malignant lesions were examined [16,18,20,21].

Concerning the diagnostic quality of non-marked CE in the differentiation between benign and malignant subpleural lung diseases, the pooled sensitivity was 0.59 (95% CI: 0.56–0.63), and the pooled specificity was 0.46 (95% CI: 0.42–0.50). The DOR was 1.10 (95% CI: 0.4–2.5). The AUC of SROC was 0.52. A significant heterogeneity was found in sensitivity (I^2^ = 96.6%), and a significant inconsistency was found in specificity (I^2^ = 86.9%) (Figure 9).

### 3.10. Diagnostic Accuracy of Early AT

**Figure 10:**
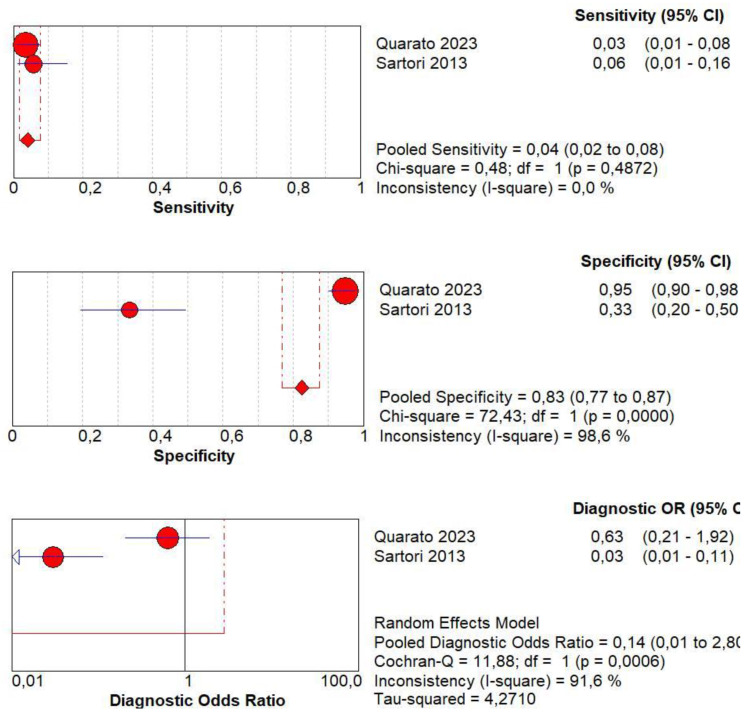

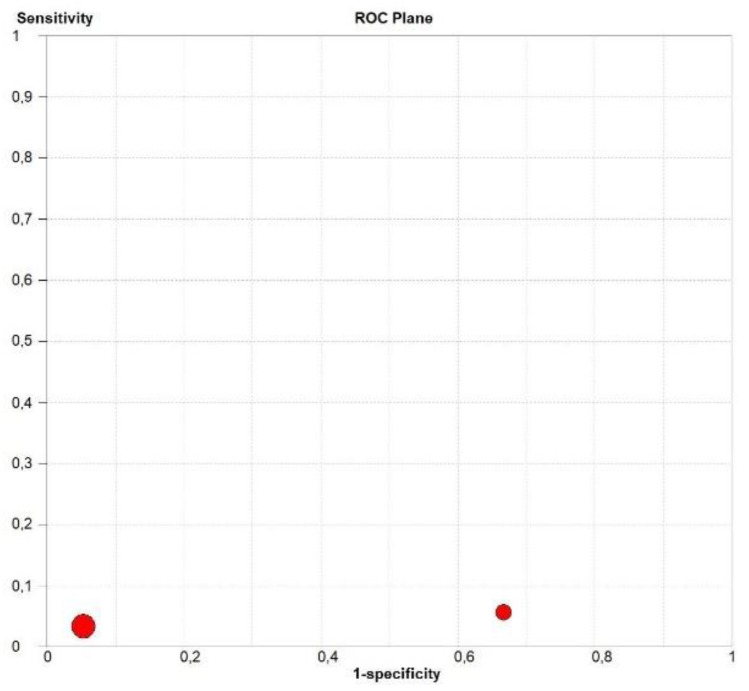
Sensitivity, specificity, diagnostic odds ratio, and SROC curve of early arrival time of contrast enhancement in differentiating malignant vs. benign peripheral lung lesions. In particular, 2 studies involving 412 patients with 200 malignant lesions were analyzed [19,20].

Concerning the diagnostic quality of early AT in the differentiation between benign and malignant subpleural lung diseases, the pooled sensitivity was 0.04 (95% CI: 0.02–0.08), and the pooled specificity was 0.83 (95% CI: 0.77–0.87). The DOR was 0.14 (95% CI: 0.0–2.8). A non-significant heterogeneity was found in sensitivity (I^2^ = 0.0%), and a significant inconsistency was found in specificity (I^2^ = 98.6%) (Figure 10).

### 3.11. Diagnostic Accuracy of Early Wash Out

**Figure 11:**
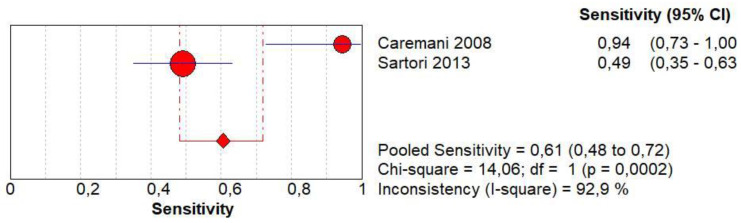

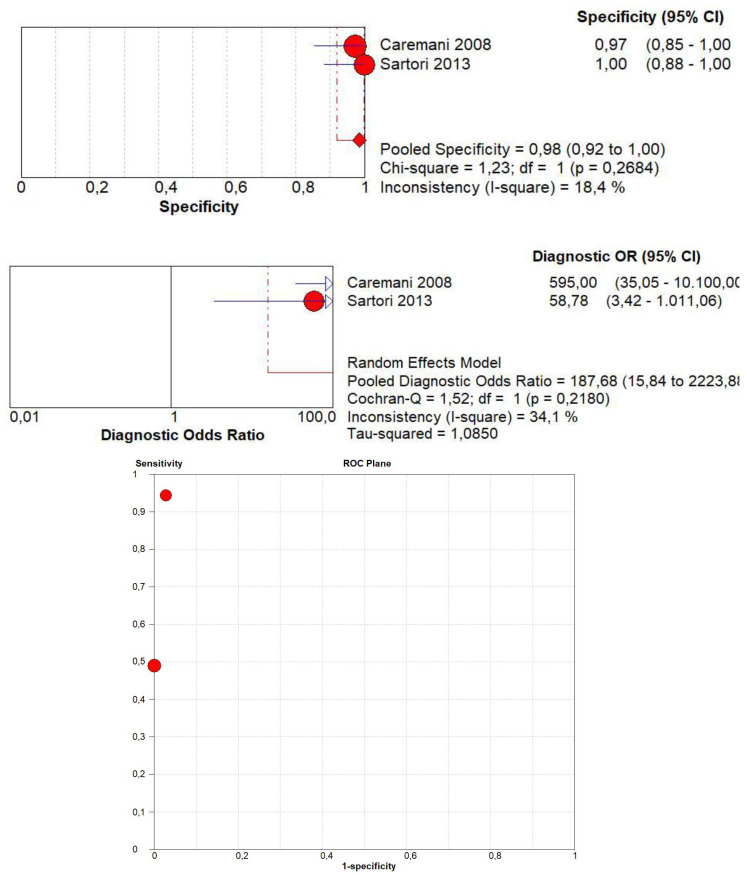
Sensitivity, specificity, diagnostic odds ratio, and SROC curve of early wash out of contrast enhancement in differentiating malignant vs. benign peripheral lung lesions. In particular, 2 studies on 155 patients with 71 malignant lesions were analyzed [17,20].

Concerning the diagnostic quality of early wash out in the differentiation between benign and malignant subpleural lung diseases, the pooled sensitivity was 0.61 (95% CI: 0.48–0.72), and the pooled specificity was 0.98 (95% CI: 0.92–1.00). The DOR was 187.6 (95% CI: 15.8–2223.8). The AUC of SROC cannot be calculated. A significant heterogeneity was found in sensitivity (I^2^ = 92.9%), and a non-significant inconsistency was found in specificity (I^2^ = 18.4%) (Figure 11).

### 3.12. Diagnostic Accuracy of Delayed Wash Out

**Figure 12:**
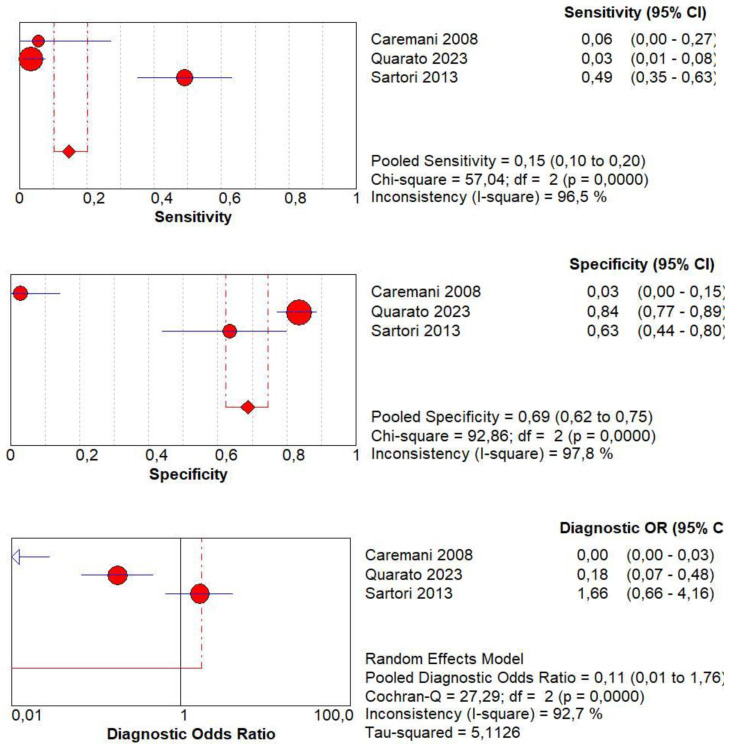

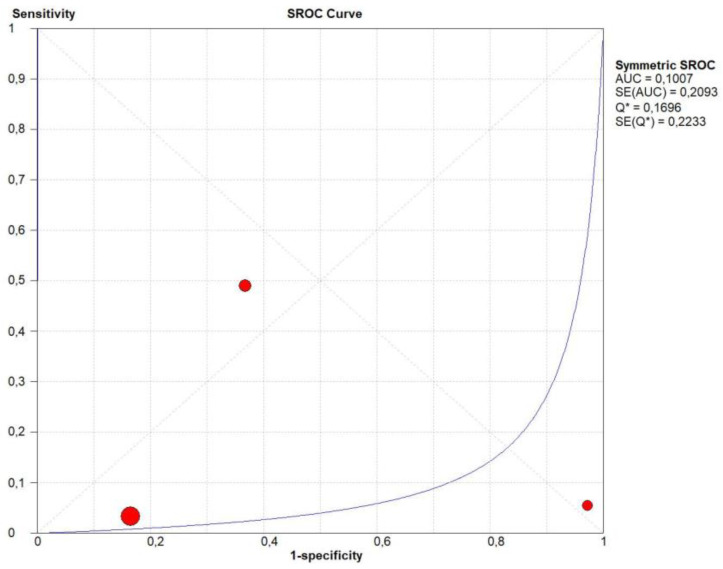
Sensitivity, specificity, diagnostic odds ratio, and SROC curve of delayed wash out of contrast enhancement in differentiating malignant vs. benign peripheral lung lesions. In particular, 3 studies on 472 patients with 218 lesions were analyzed [17,19,20].

**Figure 13:**
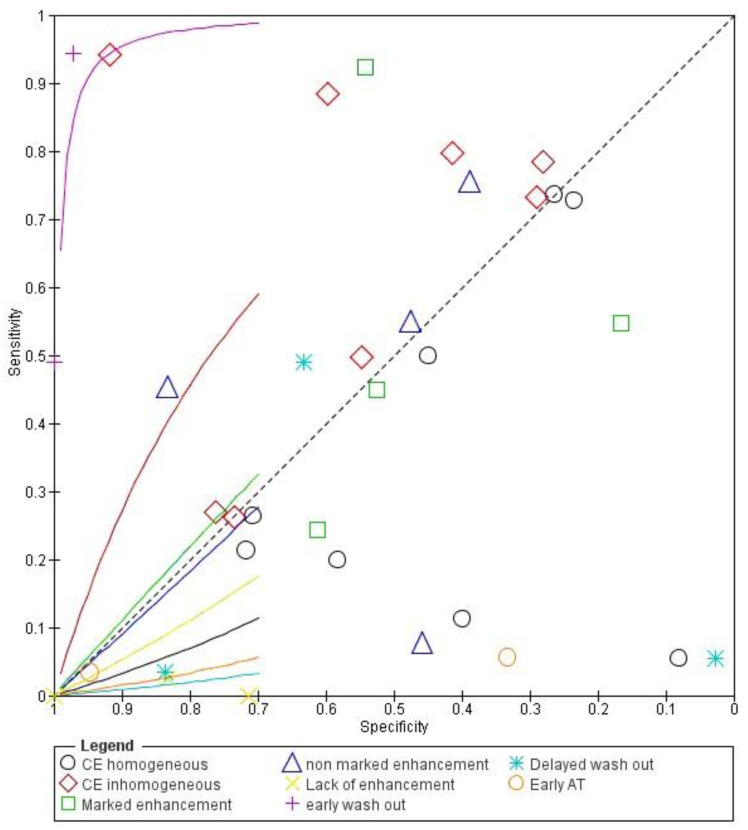
Pooled SROC curve of different contrast-enhanced ultrasound features in differentiating malignant vs. benign peripheral lung lesions. Contrast enhancement, CE; arrival time, AT.

Concerning the diagnostic quality of delayed wash out in the differentiation between benign and malignant subpleural lung diseases, the pooled sensitivity was 0.15 (95% CI: 0.10–0.20), and the pooled specificity was 0.69 (95% CI: 0.62–0.75). The DOR was 0.11 (95% CI: 0.0–1.7). The AUC of SROC was 0.10. A significant heterogeneity was found in sensitivity (I^2^ = 96.5%), and a significant inconsistency was found in specificity (I^2^ = 97.8%) (Figure 12 and Figure 13).

### 3.13. Publication Bias

**Figure 14:**
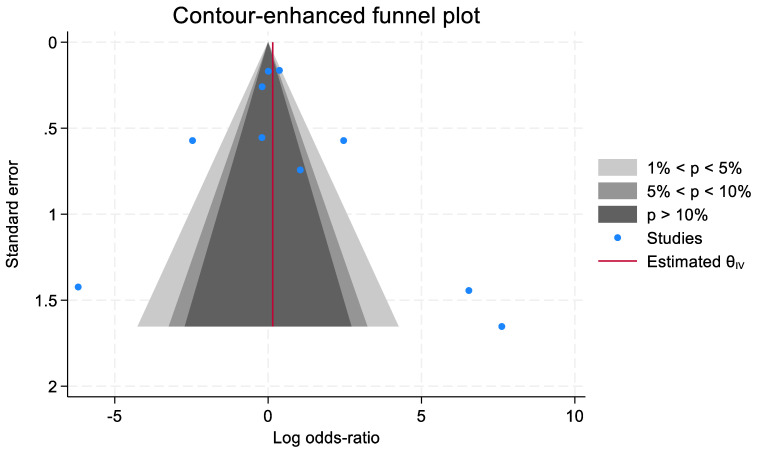
Evaluation of publication bias by contour-enhanced funnel plot.

The results of the contour-enhanced funnel plot certified that among the studies examining CEUS, no publication bias has been noted (*p* < 0.01) (Figure 14).

## 4. Discussion

In the literature, only three works provided an overall diagnostic accuracy of the CEUS method in relation to the differential diagnosis between malignant and benign lesions [16,17,22]. The meta-analysis was performed on 970 patients with 536 malignant lesions, and it showed CEUS to display a pooled sensitivity of 0.95 and a pooled specificity of 0.93 for the diagnosis of malignant lesions. It should be noted that the data on sensitivity are characterized by a significant inconsistency of 96%, while that on specificity shows a non-significant inconsistency of 4.9%. The SROC curve shows an optimal AUC of 0.97, with a DOR of 301.2.

Sperandeo et al. demonstrated that the intralesional component enhancement was consistent with cancer neovascularization [22] Moreover, unenhanced areas associated with necrotic zones were present in certain situations [22]. Twenty other lesions were investigated; these included two fibrous lung tumors, two noncaseous granulomas, one rheumatoid nodule, six abscesses, one histiocytosis X, one chondroid hamartoma, one sclerosing hemangioma, and two sarcoid nodules. All of these lesions were benign, and none of them showed intralesional enhancement [22].

In their work, Caremani et al. evaluated the enhancement of the lesion, the visibility of pulmonary arteries, and wash out, thus showing CEUS to be characterized by a sensitivity of 95.0% in comparison with that of CT (96.66%), B-Mode ultrasound (83.33%), and conventional radiology (86.66%) [17].

In the work by Bi et al., a predictive model was constructed with six parameters: the angle between lesion border and thoracic wall, basic intensity, lung lesion arrival time difference, ratio of arrival time difference, vascular sign, and non-enhancing region type [16]. The model displayed a sensitivity of 94.82% in the development cohort and 92.86% in the validation cohort and a specificity of 92.42% in the development cohort and 92.59% in the validation cohort [16].

By dividing the CEUS into the various types of contrastographic findings, the diagnostic accuracy of the individual parameters decreases; these data are also due to the fact that there are few published works. Generally, it is known that tumor lesions take contrast medium; indeed, a lesion that does not take the contrast medium is rather suggestive of a benign lesion (the lack of CE demonstrates a SROC of 0.50 and DOR of 0.07 in our analysis for malignant lesions). As anticipated in the Introduction, the impossibility of dividing the examination into the “canonical” three arterial, portal, and late phases is one of the main difficulties in applying CEUS to the study of lung lesions. Therefore, the finding of hyperenhancement in the arterial phase cannot currently be assessed. In some works, reference is made to marked or unmarked CE. In our meta-analysis, marked CE has a DOR of 0.91 (SROC: 0.47) while unmarked CE has a DOR of 1.1 (SROC: 0.52). Therefore, the two findings are not significant and indicative of a malignant lesion rather than a benign one. Subsequently, some works evaluated the homogeneity of the CE of the lesions. In general, any malignant lesion in any organ is expected to have a non-homogeneous CE due to the frequent presence of hypovascularized and/or necrotic areas. The results of our meta-analysis highlighted a DOR of 0.5 for homogeneous CE (SROC: 0.35) and 1.9 for inhomogeneous CE (SROC of 0.64).

Furthermore, two works reported the parameter defined as the early AT of contrast medium [19,20]; in the work of Quarato et al., CE AT was classified as “early” if the contrast agent reached the target lesion within 10 s [19]. In the work of Sartori et al., the AT was defined as “early” if the contrast agent reached the target lesion within 0–1 s with respect to a normal lung [20]. In our meta-analysis, early AT was characterized by a DOR of 0.14 toward malignant lesions, which is almost suggestive of benign lesions. The evaluation of wash out is one of the most relevant parameters to define the nature of a lesion. For example, at the liver level, lesions presenting wash out are usually malignant, with some exceptions such as adenomas and some cases of focal nodular hyperplasia. Furthermore, a presumptive etiological diagnosis is possible since metastases present an early wash out, while a lesion that can be referred to as HCC presents a delayed and mild washout [26,27]. By considering the findings of our systematic review, only two works specifically examine the early wash out data on lung lesions [17,20].

In the work of Sartori et al., wash out was considered “early” if the contrast medium leaves the lesion within 60 s [20]. In the work of Caremani et al., wash out was considered “early” if the contrast medium leaves the lesion within 120 s [17]. By considering the findings of our meta-analysis, early wash out is characterized by a pooled sensitivity of 0.61 and a pooled specificity of 0.98. Since the data were calculated based on two works only, the SROC cannot be calculated, but the DOR appears to be 187.6; therefore, the data were strongly associated with the diagnosis of malignancy, with a non-significant heterogeneity for the specificity of the data.

The data on delayed wash out is less significant, thus displaying a pooled sensitivity of 0.15, a pooled specificity of 0.69, and an SROC of 0.10. The DOR is 0.11; therefore, it is not indicative of malignancy. It is relevant to specify that CE wash out was defined as “delayed” if the disappearance of contrast agent from the target lesion occurred after 300 s in the work of Quarato et al. [19], after 120 s in the work of Caremani et al. [17], and after 60 s in the work of Sartori et al. [20].

Therefore, there is a clear heterogeneity in defining the different phases (early or delayed) of the CEUS study among the different published works. From the evaluation of the works analyzed in the meta-analysis, an early phase could be defined in the first 10–15 s, while a delayed phase can be defined after 60 s.

## 5. Strengths and Limitations of CEUS Characterization of Subpleural Lung Lesions

The concept of characterizing a lung lesion through thoracic ultrasound and completion with the ultrasound contrast medium is certainly fascinating. The data from this meta-analysis support and strengthen the potential of the method. Despite this, as underlined several times in this work, the works that have analyzed the global diagnostic accuracy of the method in characterizing subpleural lung lesions are only three in number, with some points of discussion (bias) such as the lack of interindividual variability or the diagnosis of reference not always performed via biopsy maneuver. A fundamental limitation of the ultrasound method to be remembered is that of the impossibility of studying “deep” lung lesions, i.e., all lesions that do not alter the pleura–lung interface. For those types of lesions, ultrasound study, including contrastography, by using EBUS could provide further diagnostic indications [28,29]. Therefore, the tomographic method with contrast medium currently remains the diagnostic gold standard [30,31,32]. CEUS in the context of transthoracic ultrasound can be used as an initial detection and screening tool for subpleural lung consolidation, but above all as a guiding tool for biopsy procedures and follow-up [33,34]. The development of quantitative CEUS methods will further facilitate the application of the method by providing more data with greater diagnostic accuracy [35].

## 6. Conclusions

The results of the meta-analysis show an excellent diagnostic accuracy of CEUS for the diagnosis of the malignancy of subpleural lung lesions (sensitivity: 95%; specificity: 93%). By dividing the CEUS method into its various findings, the diagnostic accuracy values are considerably lower and not significant in some cases. Non-homogeneous contrast enhancement with early wash out are the features that are the most indicative of the malignancy of a lung lesion. The development of quantitative CEUS, with the evaluation of more specific parameters, could increase the accuracy of the method.

## Figures and Tables

**Table 1:** Summary of findings of the studies; benign, B; malignant, M; contrast enhanced ultrasound, CEUS.

Author	Year	Study Design	Patients	Age	Male/Female	Benign	Malignant	Size of Lesions	Standard Reference	Operators	CEUS
Bai et al. [15]	2022	Retrospective observational	42	55.6	26/16	12	30	5.57 ± 1.73 cm	Biopsy	1 operator	Convex probe; Sulfur hexafluoride powder (Italian Bracco Company) was dissolved in 5 mL of 0.9% sodium chloride and immediately and fully shaken to form a suspension. For the CEUS process, 2.0 mL of the suspension was obtained and quickly pushed into the median cubital vein, followed by an injection of 5 mL of 0.9% sodium chloride.
Bi et al. [16]	2021	Retrospective and prospective	812	59.0	587/225	372	440	M: 4.65 cm (3.49–6.83) B: 4.26 cm (3.13–5.32) *p* = 0.010	Histopathology is the priority; when histopathology could not make a definite diagnosis, microbial evidence, imaging findings, clinical symptoms, and treatment effects were used. All cases were followed up with for at least 12 months.	Two operators performed this process together (with 4 and 5 years of experience in lung US). For discordant assessments, a third senior operator (with 18 years of experience of lung US) was consulted on the cases and would make the final decision	1–6 MHz convex probe; the mechanical index was set at 0.1, and the gain was adjusted to show the surface of air-filled lungs only (20 dB). Then, 1.5 mL of UCA was injected into the median cubital vein within 2s via a 20 gauge needle followed by an immediate flush with 5 mL of normal saline.
Caremani et al. [17]	2008	Prospective	60	56.0	34/26	42	18	Not specified	CT and biopsy	Not specified	3.5 MHz convex probe with a low mechanical index; SonoVue 2.4 mL bolus injection followed by a flush of saline solution.
Findeisen et al. [18]	2022	Prospective	63	64.0	46/17	24	39	M: 31.2 ± 18.9 mm B: 18.3 ± 15.1 mm *p* = 0.003	Biopsy	The evaluation of the CEUS parameters was carried out retrospectively by two independent, experienced operators based on the images. In the event of disagreement, a third investigator adjudicated.	3–6 MHz convex ultrasound probe; CEUS was performed in 1.5 MHz cadence pulse sequence mode (a contrast-specific, continuous mode software and low mechanical index).
Quarato et al. [19]	2023	Prospective	317	52.1	215/102	170	147	B: 3.2 ± 0.9 cm (1.5–8.0) M: 2.7 ± 0.5 cm (1.25–5.75) *p* = 0.0004	Biopsy or clinical and radiological follow-up	Ultrasound examination was independently performed by 2 operators with over 20 years of experience in lung ultrasonography The clips were blindly reviewed by another operator with 35 years of experience. Cohen’s k values of the diagnostic results ranged from 0.81 to 1.00, indicating almost perfect agreement between operators.	Multifrequency convex probe (3.5–5 MHz); the pre-setting for thoracic ultrasound in B-mode (i.e., gain compensation), 40–50%; dynamic range, 60–70 dB; depth, 70–140 mm; electronic imaging focus on the pleural line; tissue harmonics on) and US contrast setting (low mechanical index ≤ 0.1). Intravenous injection of 4.8 mL of SonoVue (Bracco, Milan, Italy) followed by 10 mL of regular saline.
Sartori et al. [20]	2013	Prospective	95	61.0	-	42	53	3.5 cm (range 1–12 cm).	Biopsy or clinical follow up	One of two physicians with at least 5 years of experience in CEUS examination of abdominal organs and well experienced with US of the lung. There was no concordance between the readers in TE and EW evaluation in 5/100 cases and 4/100 cases, respectively (r = 0.899, and r = 0.9, respectively). In all these cases, final consensus was reached after collegial review and discussion of the CEUS clips.	3.5 to 5.0 MHz convex transducer and a 5.0 to 7.5 MHz linear transducer; CEUS was performed with a low mechanical index contrast-specific non-linear technique and an 8 microliters/mL solution of SonoVue; acoustic power was set at 40 kilo pascal for both high-frequency linear transducer and low-frequency convex transducer.
Shen et al. [21]	2022	Retrospective observational	506	59.0	351/155	219	287	B: 4.2 ± 2.1 cm M: 5.9 ± 4.7 cm	All malignant cases were diagnosed by pathology, while benign cases were diagnosed by two respiratory physicians after comprehensive analysis of pathology, etiology, imaging, and clinical symptoms.	The imaging data were blindly reviewed by 2 experienced operators in pulmonary CEUS examinations. If the two radiologists agreed on a diagnosis, the final result was the same; if they disagreed, a third senior radiologist analyzed it, and they all discussed it together.	2.8–5.0 MHz convex probe; The mechanical index was adjusted to 0.10, the total gain was 20, and the dynamic range was 69 DB; SonoVue solution (1.5 mL) was administered by bolus injection via the antecubital vein, followed by 5 mL of saline.
Sperandeo et al. [22]	2006	Prospective	98	60.0	65/33	20	78	-	Biopsy	Single physician experienced with ultrasonography of the lung	Multifrequency convex array transducers (3.5 MHz); SonoVue 4.8-mL bolus was administered via a 20-gauge IV cannula in an antecubital vein. The injection was immediately followed by a 10-mL bolus of 0.9% sodium chloride. The CEUS scan was performed in the harmonic mode with a mechanical index of 0.04 or less.
Sperandeo et al. [23]	2017	Prospective	728	65.0	-	329	399	-	Clinical course, imaging, and laboratory and/or histology test	A single physician with 25 years of experience in lung ultrasonography performed and digitally recorded all CEUS scans. The clips were blindly reviewed by another operator with 20 years of experience. Inter-reader agreement was excellent (Spearman’s coefficient ≥ 0.90 for all parameters)	Multifrequency (3.5–5 MHz and 3–8 MHz) convex probe; SonoVue bolus of 4.8 mL of Sonovue followed by 10 mL regular saline was injected Intravenously; a CEUS scan was performed with a mechanical index of ≤0.04.
Tang et al. [24]	2020	Retrospective	96	61.7	71/25	45	51	B: 5.54 cm (0.26) M: 7.71 cm (0.40) *p* < 0.001	Biopsy	Two operators with 5 years’ experience in ultrasound diagnosis who were blinded to the pathological diagnosis of the patient, observed the CEUS analysis together and reached an agreement	3.5–5 MHz probe SonoVue 2.4 mL were rapidly injected via the elbow vein, followed by a rapid bolus injection of 5 mL normal saline.

## Data Availability

The data can be requested from the corresponding author.
